# Notes of Treatment by Prof. Da Costa

**Published:** 1885-12-20

**Authors:** 


### NOTES OF TREATMENT BY PROFESSOR DA COSTA.

BY THE REPORTER OF "CLASS-ROOM NOTES."

### TREATMENT OF PULMONARY CONSUMPTION.

*Hygienic Treatment.*—Out-door exercise, good food, warm clothing ; climate of paramount importance. The best climate, by far, is that found in Egypt ; Algeria is a good place. In this country, New Mexico, Southern California, South Carolina, Thomasville in Georgia, Florida. Colorado, for some cases, is an excellent climate. Cases having a co-existing bronchitis do better in a damp and mild climate, as Florida, etc. The element of change is very useful. The Adirondacks is a fine place for those early cases in which there is no tendency to hemorrhage. Professor Da Costa does not care much for the "mild diet," but allows it in conjunction with other things. Give plenty of meats, and alcohol in moderation, especially in those cases free from fever. Mix it with ol. morrhuæ, to lessen the tendency to its abuse. Whisky and brandy are the best stimulants here. You need not interdict smoking.

*Medicines.*—Ol. morrhuæ is of great utility by improving nutrition and also by affecting the tubercle. Do not use its substitutes, as glycerine, etc. Give f ℥ss. ter die, one hour after meals. To disguise it, and to promote its ready absorption, give *m* x–xv ether, but this sometimes causes belching. Mix it with equal amount of malt or whisky. When the apetite fails stop its use for a while. Do not permit the oil to be taken in hot weather.

Next in importance is arsenic in small doses in the early stages; arsenous acid, gr. 1-40 or gtt. iij Fowler's solution, ter die. In the late stages it will be of no avail.

A third remedy is iodine ; it should be more generally used ; liq. iodi comp. gtt. i–iij, ter die, with potassium iodide to alternate with it. When anæmia is present, and not much fever, use iodide of iron. It is very valuable. Push it up to the point of tolerance. Begin with gtt. xv of the official syrup, and push up to f℥j, ter die.

Professor Da Costa does not like the hypophosphites. They have no special effect, as ol. morrhuæ and arsenic. Inhalations of sodium benzoate are of no use. Carbolic acid and tar by inhalation are of some avail.

*Treatment of Special Symptoms.*—Entirely too much is done for the symptoms. For cough we should give no expectorant unless bronchitis exists. Since the cough is generally an irritative one, morphia must, in time, be given. Codeia, gr. $\frac{1}{8}$–$\frac{1}{4}$, in simple elixir, often has a wonderful effect, and does not constipate. Prussic acid or fluid extract of wild cherry is very useful at times. We may combine the acid with morphia. Inhalations of oil of eucalyptus give relief.

*Night Sweats.*—Give atropia, gr. 1-80, at bedtime. Sponge off the body with hot water to constringe the vessels. Infusion of sage at night. Mineral acids, especially sulphuric acid. Zinc oxide, gr. ij, ter die. Ergotin or fluid extract of ergot is better than morphia in some respects. It is more permanent and does not cause dryness. Give ergotin, gr. ij, ter die, the last dose at bedtime.

*Digestive System.*—The patient often has vomiting. Two excellent remedies may be given, as carbolic acid or creasote, gr. $\frac{1}{4}$, four times per diem. Strychnia, gr. 1-50, ter die, is also of great value.

*Diarrhœa.*—Opium, $\frac{1}{4}$gr.; bismuth, ℈j; copper sulphate, gr. 1-12; silver nitrate, gr. $\frac{1}{4}$, etc.

*The Throat in Phthisis.*—It may be swollen, and the larynx the seat of ulcers, which may become tubercular. Drink demulcents, as Irish moss (ʒj to the Oj).

Professor Da Costa has confidence in local applications of iodoform and cocaine. Let the patient eat his meals while the parts are under the effect of cocaine.

*For Irritative Fever—*

℞. Quininæ sulph.... ........................gr. iss,
Digitalis,..................................gr. ss,
Opii.......................................gr. $\frac{1}{4}$.
M. Ft. pil.
Sig: Ter die.—*College and Clinical Record.*

